# Differential DNA methylation 7 months after SARS-CoV-2 infection

**DOI:** 10.1186/s13148-025-01866-4

**Published:** 2025-04-18

**Authors:** Peizhen Hong, Melanie Waldenberger, Michael Pritsch, Leonard Gilberg, Isabel Brand, Jan Bruger, Jonathan Frese, Noemi Castelletti, Mercè Garí, Christof Geldmacher, Michael Hoelscher, Annette Peters, Pamela R. Matías-García, ORCHESTRA-study group

**Affiliations:** 1 Research Unit of Molecular Epidemiology, Institute of Epidemiology, Helmholtz Zentrum München, German Research Center for Environmental Health, Neuherberg, Germany; 2 Institute of Epidemiology, Helmholtz Zentrum München, German Research Center for Environmental Health, Neuherberg, Germany; 3 Institute for Medical Information Processing, Biometry, and Epidemiology (IBE), Faculty of Medicine, Ludwig-Maximilians-Universität (LMU) Munich, Munich, Germany; 4 Pettenkofer School of Public Health, Munich, Germany; 5 German Centre for Cardiovascular Research (DZHK), Partner Site Munich Heart Alliance, Munich, Germany; 6 Institute of Infectious Diseases and Tropical Medicine, LMU University Hospital, LMU Munich, Munich, Germany; 7 German Center for Infection Research (DZIF), Partner Site Munich, Munich, Germany; 8 Department of Infectious Diseases, LMU University Hospital, LMU Munich, Munich, Germany; 9 Division of Clinical Pharmacology, Department of Medicine IV, LMU University Hospital, LMU, Munich, Germany; 10 Institute of Computational Biology, Helmholtz Zentrum München, German Research Center for Environmental Health, Neuherberg, Germany; 11 Center for International Health (CIH), University Hospital, LMU Munich, Munich, Germany; 12 Immunology, Infection and Pandemic Research, Fraunhofer Institute for Translational Medicine and Pharmacology ITMP, 80799 Munich, Germany

**Keywords:** COVID-19, SARS-CoV-2, Epigenome-wide association study, DNA methylation

## Abstract

**Background:**

Severe acute respiratory syndrome coronavirus 2 (SARS-CoV-2) causes coronavirus disease 2019 (COVID-19), and SARS-CoV-2 has been linked to changes in DNA methylation (DNAm) patterns. Studies focused on post-SARS-CoV-2 infection and DNAm have been mainly carried out among severe COVID-19 cases or without distinguishing the severity of cases. However, investigations into mild and asymptomatic cases after SARS-CoV-2 infection are limited. In this study, we analyzed DNAm patterns of mild and asymptomatic cases seven months after SARS-CoV-2 infection in a household setting by conducting epigenome-wide association studies (EWAS).

**Results:**

We identified DNAm changes at 42 CpG sites associated with anti-SARS-CoV-2 antibody levels. We additionally report EWAS between COVID-19 cases and controls, with the case status being confirmed by either an antibody test or a PCR test. The EWAS with an antibody test case definition identified 172 CpG sites to be differentially methylated, while the EWAS with a PCR test case definition identified 502 CpG sites. Two common sites, namely cg17126990 (annotated to *AFAP1L2*) and cg25483596 (annotated to *PC*), were identified to be hypermethylated across the three EWAS. Both CpG sites have been reported to be involved in molecular pathways after SARS-CoV-2 infection. While *AFAP1L2* has been found to be upregulated after SARS-CoV-2 infection, the pyruvate carboxylase (PC) activity seems to be affected by SARS-CoV-2 infection resulting in changes to the host cell metabolism. Additionally, an EWAS to assess persistent health restrictions among PCR-confirmed cases showed 40 CpG sites to be differentially methylated.

**Conclusions:**

We detected associations between DNAm in individuals who had asymptomatic and mild SARS-CoV-2 infections as compared to their household controls. These findings contribute to our understanding of the molecular consequences of SARS-CoV-2 infection observed months after infection.

**Supplementary Information:**

The online version contains supplementary material available at 10.1186/s13148-025-01866-4.

## Introduction

Coronavirus disease 2019 (COVID-19) has brought huge health challenges to the world; as of August 2023, more than 760 million cases and 6.9 million deaths have been documented worldwide [1]. Infected patients have a variety of clinical manifestations, from being asymptomatic or having mild symptoms, to severe illness including acute respiratory failure, septic shock, and multiple organ failure [2]. Most people who get sick with COVID-19 will recover without hospital treatment; however, the elderly, males, and those with pre-existing health conditions tend to have severer symptoms [3, 4]. Some people who have recovered from the initial COVID-19 experience long-lasting symptoms including fatigue, breathlessness, and cognitive dysfunction, which is known as post-COVID-19 condition [5].

Severe acute respiratory syndrome coronavirus 2 (SARS-CoV-2) is the virus that causes COVID-19. Spike proteins on the surface of the SARS-CoV-2 mediate the host cells entry. The S1 subunit of the spike proteins binds to host entry receptor angiotensin-converting enzyme 2 (ACE2), and the S2 subunit mediates membrane fusion where spike proteins are cleaved by type 2 transmembrane serine protease (TMPRSS2) [6]. ACE2 and TMPRSS2 are widely expressed in lungs, hearts, kidneys, and multiple organs which intrigue the specific clinical manifestation of COVID-19 [7]. Both innate and adaptive immune responses are involved in the inflammatory response of the host defense, with increased viral loads leading to the activation and proliferation of immune cells, the production of proinflammatory and anti-inflammatory cytokines, ultimately resulting in various destructive events [8, 9].

DNAm is a molecular mechanism involved in the regulation of gene expression without changing the underlying DNA sequence. Methylation most often occurs in cytosine-phosphate-guanine (CpG) dinucleotides, where a methyl group is transferred to the C-5 position of the cytosine ring by DNA methyltransferases. DNAm plays a crucial role in normal development; its dysregulation is involved in the onset and progression of several human diseases. Therefore, DNAm changes are increasingly considered as diagnostic and prognostic biomarkers in clinical practice, for example, in human cancers [10].

Epigenetic regulation also plays an important part in the pathophysiology of COVID-19, as SARS-CoV-2-infected patients were reported to have DNAm alterations [11]. SARS-CoV-2 affects DNAm patterns that regulate *ACE2* gene expression, which in turn is associated with the susceptibility to COVID-19 [12]. Studies also found that DNAm impacts COVID-19 severity by regulating the immune response [13, 14]. Differentially methylated regions were identified in blood samples of hospitalized individuals one year after recovering from acute illness [15]. An epigenome-wide association study (EWAS) conducted in the Norwegian Corona Cohort, which included both mild and severe COVID-19 patients, also found DNAm changes three months after infection [16].

A complete understanding of persistent DNAm variation in mild and asymptomatic cases after SARS-CoV-2 infection is missing. To better understand the molecular mechanisms underlying COVID-19, the aim of this study was to examine DNAm associations seven months post-SARS-CoV-2 infection in a household setting.

## Methods

### Study population

The prospective COVID-19 cohort Munich (KoCo19) was launched in 2020 in Munich, Germany. KoCo19-Shield was a substudy of KoCo19 to investigate SARS-CoV-2-specific immune responses in convalescent individuals more than 3 months after infection. In KoCo19-Shield, households with at least one person who had a polymerase chain reaction (PCR)-confirmed SARS-CoV-2 infection were recruited, as described in detail in a prior publication [17]. From September 2020 to January 2021, 177 PCR-positive individuals and 145 of their household members from 157 households were enrolled, either through house visits or at the study center at the Division of Infectious Diseases and Tropical Medicine, University Hospital, LMU Munich. In January 2021, 85 members of 36 households from KoCo19 were randomly selected as controls, all of them consistently tested negative for SARS-CoV-2-specific antibodies throughout the first year of the COVID-19 pandemic [18, 19]. All participants including PCR-confirmed individuals, their household members, and controls were asked to provide a venous blood sample with additional information about the course of the disease and their living situation. Personal and clinical data from participants were collected using the mobile data collection tool OpenDataKit via Android smartphones [20].

### DNAm analysis

Genomic DNA (750 ng) from 382 individuals was bisulfite converted using the EZ-96 DNA Methylation Kit (Zymo Research, Orange, CA, USA). Subsequent methylation analysis was performed on an Illumina (San Diego, CA, USA) iScan platform using the Infinium MethylationEPIC BeadChip v1 according to standard protocols provided by Illumina. GenomeStudio software version 2011.1 with Methylation Module version 1.9.0 was used for initial quality control of assay performance and for generation of methylation data export files. Further quality control and preprocessing of the data were performed in R v4.1.3 [21] with the package minfi v1.40.0 [22] and following primarily the CPACOR pipeline [23]. Raw intensities were read into R and background corrected. Probes with detection *p*-values > 10^{− 16} were set to missing and were retained as missing in all subsequent QC steps.

Before normalization, 4 samples were removed, as they had failed the sex prediction or the median intensity quality control steps or had > 20% missing values on the autosomes. A total of 68,017 probes were removed (some overlapping multiple categories): cross-reactive probes as given in published lists (*N* = 44,493); probes with SNPs with minor allele frequency > 5% at the CG position (*N* = 11,370) or the single base extension (*N* = 5,597) as given by minfi; and 15,667 with > 5% missing values (autosomes only). CpGs from the EPICv1 no longer appearing in the EPICv2 and CpGs from X and Y chromosomes were additionally removed, yielding a total of 690,738 available for analysis (79.77% of the probes).

Quantile normalization (QN, R package limma v3.50.3 [24]) was then performed separately on the signal intensities divided into the 6 probe types: type II red, type II green, type I green unmethylated, type I green methylated, type I red unmethylated, type I red methylated [23]. For the autosomes, QN was performed for all samples together. The transformed intensities were then used to generate methylation beta values, a measure from 0 to 1 indicating the percentage of methylated bead-type intensity to the total locus intensity.

### Serology assessment

SARS-CoV-2 antibody reactivity was measured in plasma derived from ethylenediaminetetraacetic acid (EDTA)-coated blood tubes using Elecsys® Anti-SARS-CoV-2 (Roche, Mannheim, Germany). A threshold of 0.422 (instead of 1.0) for anti-SARS-CoV-2 antibody was used to determine seropositive in the participants [25].

**Fig. 1:**
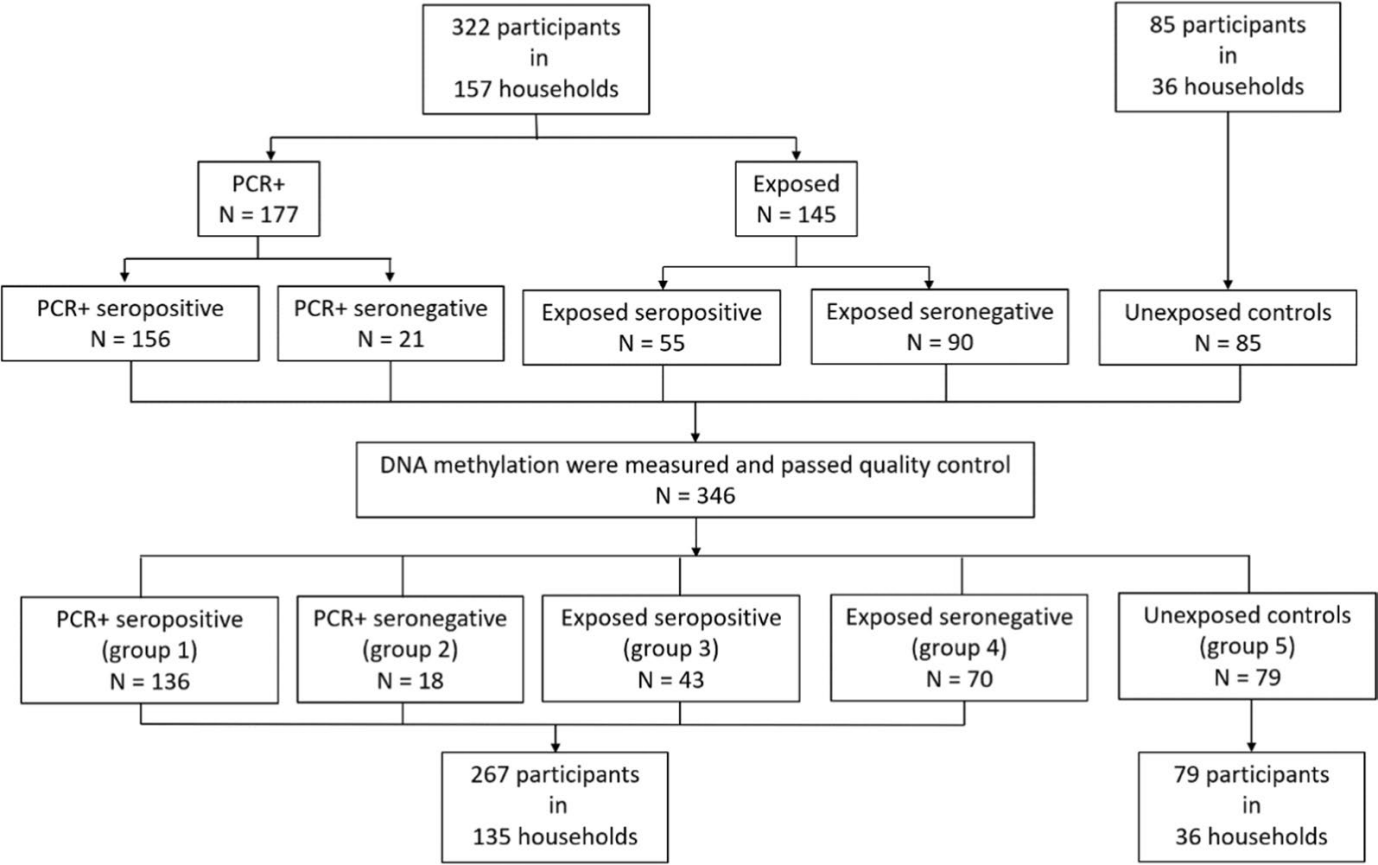
Flow diagram of the study population

In total, 346 participants with both clinical data and DNAm data were included in the analysis (Fig. 1). The study subjects were then categorized into five groups: 136 individuals who were PCR positive and seropositive (group1), 18 people who were PCR positive and seronegative (group2), 43 participants who were exposed household members and seropositive (group3), 70 individuals who were exposed members and seronegative (group4), and 79 individuals who were seronegative controls (group5). The case and non-case classification of antibody testing was not completely consistent with that of PCR testing, and the exposed household members with seropositive results were not confirmed by a PCR test. Therefore, in our subsequent analyses, anti-SARS-CoV-2 antibody levels, COVID-19 status based on antibody testing (group1 + group3 VS group2 + group4 + group5), and COVID-19 status based on PCR testing (group1 + group2 VS group4 + group5) were both analyzed as variables of interest.

### Statistical analysis

Data analysis was conducted using R 4.3.1 [21]. An epigenome-wide association study approach using generalized estimating equation (GEE) models with an exchangeable correlation matrix and grouping factor households was applied to explore the relation between COVID-19 phenotypes and DNAm. DNAm outliers that were not within the range from 25th percentile−3*interquartile range (IQR) to 75th percentile + 3*IQR were removed, leading to the removal of 0.3% of the data points. The methylation beta-value was transformed to M-value as dependent variable, as it is more statistically valid and provides less biased results [26, 27]. To examine the impact of COVID-19, variables/phenotypes indicating anti-SARS-CoV-2 antibody levels, COVID-19 status based on antibody testing, and COVID-19 status based on PCR testing were included in the model, respectively. Additional variables included in the models to control for confounding were age, sex, CD8 + T-lymphocytes, CD4 + T-lymphocytes, natural killer cells, B cells, and monocytes. Granulocytes were excluded as a covariate to avoid multicollinearity.

To examine the long-term effect of COVID-19, PCR-confirmed individuals were divided into two groups and analyzed using the same model. The classification was based on participants’ responses to the question “consequence of illness from COVID-19.” Those who reported persistent health restrictions or long-term consequences were categorized into the persistent health restriction group, while those who reported recovery were categorized into the recovery group.

Sensitivity analysis included additional adjustment for smoking. Due to missingness and poor quality of self-reported smoking status, this variable was obtained by the classifier EpiSmokEr, which inferred smoking status based on 121 CpGs [28]. Although there were only 100 overlapping CpGs in our data, the performance of the classifier was validated using data from KORA F4, which resulted in a sensitivity of 71.1% and a specificity of 94.3% to discriminate current and non-current smokers. Another subset of participants was also considered in a secondary sensitivity analysis: This subset excluded 4 PCR-confirmed cases with no blood testing dates and their 5 household members, as well as 1 case for whom the interval between PCR testing and blood sample collection was only 19 days and one additional member of this household.

To identify the biological pathways, differentially methylated CpG sites of three models were mapped to Entrez Gene IDs and tested for Gene Ontology (GO) and Kyoto Encyclopedia of Genes and Genomes (KEGG) pathways by R package missMethyl [29].

All analyses were performed on complete data for all variables included in the model. Bacon method was used to control inflation by applying the empirical null distribution [30]. False discovery rate (FDR) correction method by Benjamini–Hochberg was applied to address multiple testing problems, with a significant set of *p* < 0.05 [31]. Findings were compared with previous published EWAS results and databases like the EWAS catalog [32]. The publicly accessible database “Genetics of DNA Methylation Consortium” (GoDMC) was used to extract cis- and trans-methylation quantitative trait loci (meQTL) and expression quantitative trait methylation (eQTM) [33–35].

## Results

### Characteristics of the study population

A total of 346 individuals from 171 households were considered in the study (Table 1). None of the PCR-confirmed COVID-19 cases was hospitalized, and they presented a mild course of illness or reported asymptomatic. The average age of all study participants was 42 years old, and the median age ranged from 35 to 42 years across groups. Forty-nine percent of study participants were females, and the proportion of females ranged from 45% to 67% across groups. The median days from PCR testing to blood sample collection were 242 days.

**Table 1:** Characteristics of participants according to COVID-19 status groups

	PCR-positive seropositive (group 1)	PCR-positive seronegative (group 2)	Exposed seropositive (group 3)	Exposed seronegative (group 4)	Unexposed controls (group 5)	All study subjects
n	136	18	43	70	79	346
*Sex*						
Female	61 (45%)	12 (67%)	24 (56%)	33 (47%)	39 (49%)	169 (49%)
Male	75 (55%)	6 (33%)	19 (44%)	37 (53%)	40 (51%)	177 (51%)
*Age (years)*						
14—19	1 (0.7%)	0 (0%)	6 (14%)	5 (7.1%)	10 (13%)	22 (6.4%)
20—34	26 (19%)	6 (33%)	14 (33%)	24 (34%)	14 (18%)	84 (24.3%)
35—49	62 (46%)	7 (39%)	10 (23%)	23 (33%)	33 (42%)	135 (39%)
50—64	37 (27%)	3 (17%)	8 (19%)	14 (20%)	15 (19%)	77 (22.3%)
65—79	10 (7.4%)	2 (11%)	5 (12%)	4 (5.7%)	3 (3.8%)	24 (6.9%)
80 +	0 (0%)	0 (0%)	0 (0%)	0 (0%)	4 (5.1%)	4 (1.2%)
Median	43	39.5	35	40	44	41
*BMI (kg/m*^2^*)*						
< 18.5	1 (0.7%)	0 (0%)	2 (4.7%)	3 (4.3%)	2 (2.5%)	8 (2.3%)
18.5—25	80 (59%)	13 (72%)	27 (63%)	43 (61%)	42 (53%)	205 (59.2%)
25—30	47 (35%)	5 (28%)	7 (16%)	22 (31%)	23 (29%)	104 (30.1%)
> 30	8 (5.9%)	0 (0%)	7 (16%)	2 (2.9%)	12 (15%)	29 (8.4%)
Median	24.1	24.4	22.8	22.8	24.2	23.7
*Smoking status*						
Current smoker	21 (15.4%)	3 (16.7%)	1 (2.3%)	10 (14.3%)	13 (16.5%)	46 (13.3%)
Non-current smoker	115 (84.6%)	15 (83.3%)	42 (97.7%)	60 (85.7%)	66 (83.5%)	300 (86.7%)
*Time from PCR to visit*						
Median (days)	243	233				
IQR (days)	229.5–258	227.8–245				

Age and BMI were divided into groups and presented medians. All categorical variables were presented as numbers (percentages)

Based on SARS-CoV-2 antibody testing, the median age of the case group (group 1 + group 3) was 41 years, the median body mass index (BMI) was 23.77 kg/m^2^, 47% were females, and 12.2% were current smokers; the median age of the control group (group 2 + group 4 + group 5) was 42 years, the median BMI was 23.51 kg/m^2^, 50% were females, and 15.6% were current smokers. Based on PCR testing, the cases group (group1 + group2) had a median age of 41.5 years and a median BMI of 24.24 kg/m^2^, with females making up 47.4% and 15.6% being current smokers, while the control group (group4 + group5) had a median age of 43 years, a median BMI of 23.36 kg/m^2^, with a proportion of female 48.3% and 15.4% current smoker. The Wilcoxon rank sum test for age and BMI and the Chi-square test for sex and smoking status all showed that the study population was well balanced across case–control groups.

Of the PCR-confirmed cases, 121 (83%) reported recovery from the illness, with a balanced ratio of median age, BMI, and sex in both groups (Table 2).

**Table 2:** Characteristics of PCR-positive individuals with different post-infection health statuses

		Persistent health restriction	Recovery	All PCR-confirmed subjects
n				
PCR-positive seropositive	21 (88%)		106 (88%)	127 (88%)
PCR-positive seronegative	3 (13%)		15 (12%)	18 (12%)
Sex				
Female	12 (50%)		56 (46%)	68 (47%)
Male	12 (50%)		65 (54%)	77 (53%)
Age in years (median [IQR])	43 [40, 48]		41 [35, 51]	41 [35, 51]
BMI kg/m2 (median [IQR])	23.96 [21.74, 26.60]		24.25 [21.51, 26.23]	24.22 [21.60, 26.32]
Time from PCR to visit days (median [IQR])	243 [223.5, 259.5]		241 [230.8, 256.3]	242 [229.5, 257.5]

All continuous variables were presented as median and interquartile range (IQR). All categorical variables were presented as numbers (percentages)

### DNAm changes associated with anti-SARS-CoV-2 antibody levels

**Fig. 2:**
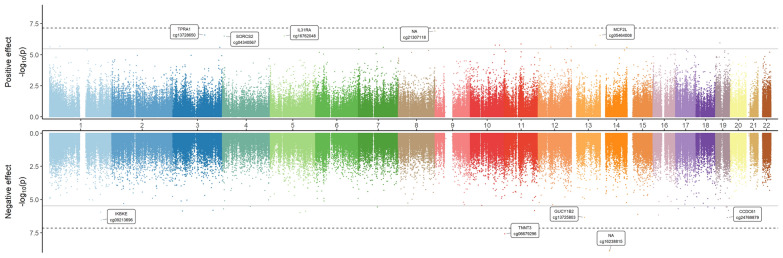
Miami plot displaying EWAS results for anti-SARS-CoV-2 antibody level (*n* = 346). X-axis represents the position of the CpGs along the chromosomes, and y-axis represents -log10 (*p*-value). The dash black line represents the Bonferroni threshold (7.24e-08). The gray line represents the FDR threshold (3.35e-06). The top panel shows positive effect sizes, while the bottom panel shows negative effect sizes. The top 5 significant CpG sites in each panel and their annotated genes are shown in boxes

The Miami plot illustrates the EWAS results for DNAm in relation to anti-SARS-CoV-2 antibody levels (Fig. 2). After inflation correction, the inflation factor lambda was 1.357 (Supplementary file 1, Figs. 1A and 2A) [30]. Forty-two differentially methylated CpG sites were identified to be associated with antibody levels at FDR < 0.05 (Supplementary file 2, Table S1). Among the significant CpG sites, 18 (43%) were hypermethylated. In addition, the results were compared with independent top cis- and trans- meQTLs and eQTMs. Three out of 42 CpGs (cg02942825 annotated to *GIPR*, cg10118093 annotated to *PIK3C2B*, cg26589785 annotated to *ITPKA*) were found to be cis-meQTLs and cis-eQTMs, suggesting that genetic variants might contribute to the regulation of local gene expression.

### Differential DNAm according to COVID-19 status

**Fig. 3:**
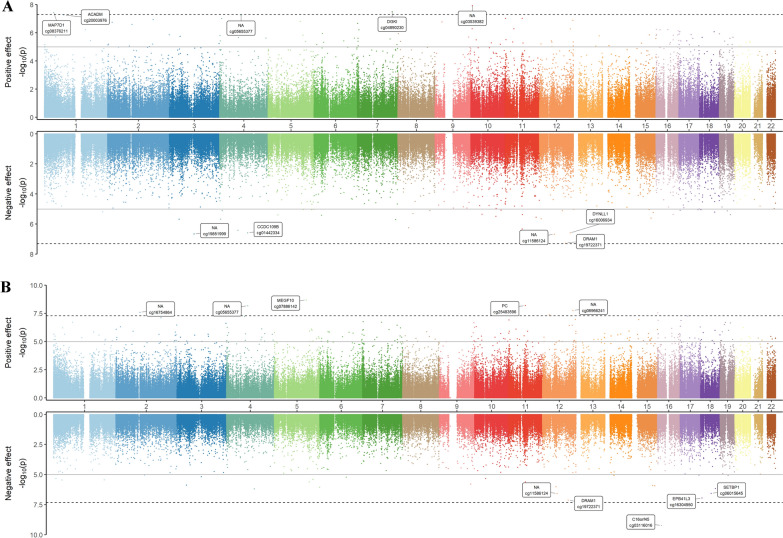
Miami plot of EWAS results between cases and controls. X-axis represents the position of the CpGs along the chromosomes, and y-axis represents -log10 (*p*-value). The top panel shows positive effect of EWAS, while the bottom panel shows negative effect of EWAS. The top 5 significant CpG sites in each panel and their annotated genes are shown in boxes. The dash black line represents the Bonferroni threshold (7.24e-08). (A) EWAS between antibody testing cases and controls (*n* = 346), the gray line represents the FDR threshold (1.34e-05). (B) EWAS between PCR testing cases and controls (*n* = 305), the gray line represents the FDR threshold (3.90e-05)

DNAm patterns between COVID-19 cases and controls were also examined. The Miami plot displays the EWAS results between COVID-19 cases and controls based on different case definitions (antibodies test VS PCR test) (Fig. 3). The Bacon correction to control for inflation and bias resulted in an inflation factor of 1.118 (Supplementary file 1, Figs. 1B and 2B) and 1.265 (Supplementary file 1, Figs. 1C and 2C) for the two approaches, respectively. The EWAS on anti-SARS-CoV-2 antibody levels showed 172 differentially methylated CpG sites (Supplementary file 2, Table S2), of which 125 (73%) were hypermethylated; 22 (13%) CpGs had a cis-meQTL, but none had cis-eQTM, thus indicating variation in genetic sequence to be associated with variation in DNAm level in 13% of the identified CpGs. In the analysis of cases and controls based on the PCR test results, 502 CpG sites were found to be differentially methylated (Supplementary file 2, Table S3). Of these, 428 (53%) were hypermethylated and 2 CpGs (cg20307496 in gene *CDC42EP1*, cg22950153 in gene *IGDCC3*) were found in both cis-meQTL and eQTM, suggesting that genetic variants might regulate local gene expression by DNAm variation.

### Persistent health restriction in PCR-positive patients

**Fig. 4:**
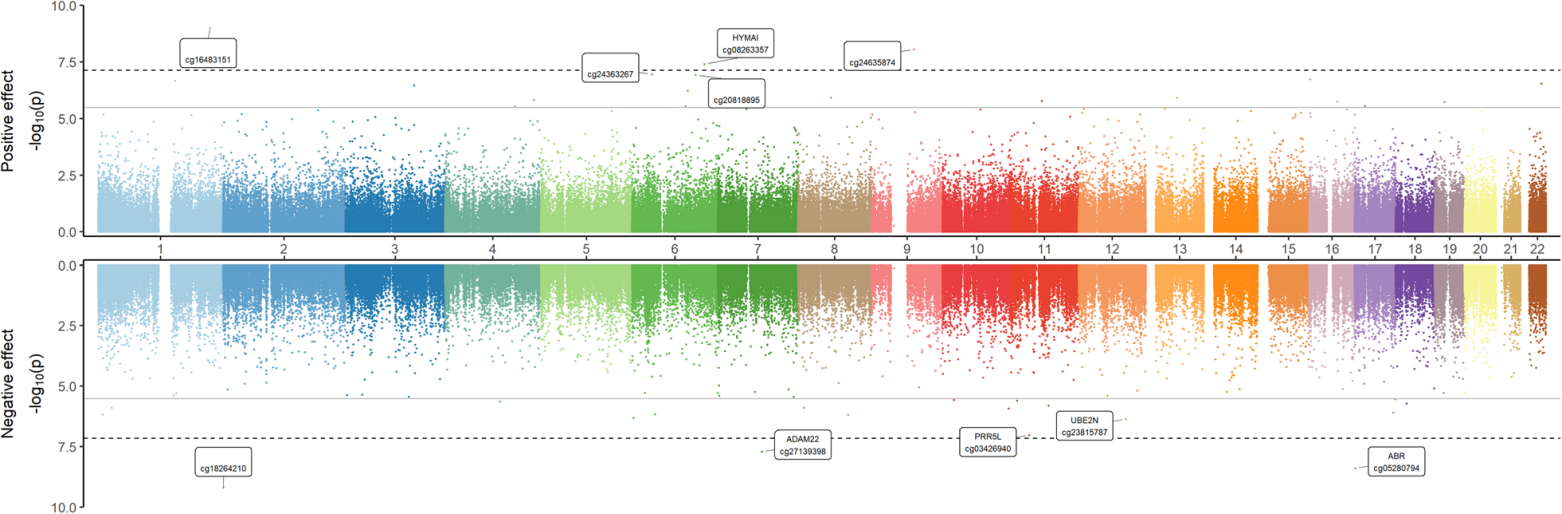
Miami plot of EWAS results between persistent health restriction and recovery cases (*n* = 139)

By investigating the long-term effect of SARS-CoV-2 infection, we found 40 differentially methylated CpG sites between persistent health restriction cases and recovery cases (Supplementary file 2, Table S4), 21 (53%) of which were hypermethylated (Fig. 4).

X-axis represents the position of the CpGs along the chromosomes, and y-axis represents -log10 (*p*-value). The dash black line represents the Bonferroni threshold (7.24e-08). The gray line represents the FDR threshold (3.15e-06). The top panel shows positive effect of EWAS, while the bottom panel shows negative effect of EWAS. The top 5 significant CpG sites in each panel and their annotated genes are shown in boxes.

### Common CpG sites across the findings

Comparing the results of the different models, two CpG sites, cg17126990 and cg25483596, both hypermethylated, appeared in all three models (Table 3). Cg17126990 was annotated to gene *AFAP1L2* and is located on chromosome 10. Another CpG site, cg25483596, annotated to the gene *Pyruvate carboxylase* (*PC*), is located within intron 10 of the gene on chromosome 11. Additionally, cg03498173 was differentially methylated in both the antibody-level association EWAS and the EWAS between antibody testing cases and controls, while cg13725803 and cg16238815 were differentially methylated CpG sites identified in both the EWAS on antibody levels and the EWAS on the PCR-based case status (Table 3).

**Table 3:** Common significant CpGs across the three EWASs

CpG	Chr	Position	UCSC RefGene Name	SARS-CoV-2 antibody level		Cases VS Controls (based on antibody testing)		Cases VS Controls (based on PCR testing)	
				Effect size	Adjusted *p*-value	Effect size	Adjusted *p*-value	Effect size	Adjusted *p*-value
cg17126990	10	116,061,880	*AFAP1L2*	0.001166	1.73E-06	0.128129	6.39E-06	0.143537	5.31E-06
cg25483596	11	66,632,197	*PC*	0.001467	1.35E-06	0.178683	9.31E-08	0.206175	6.28E-09
cg03498173	12	89,746,771	*DUSP6*	− 0.00199	1.78E-06	− 0.17119	7.85E-06	–	–
cg13725803	13	51,640,297	*GUCY1B2*	− 0.00153	4.59E-07	–	–	-0.13815	2.97E-05
cg16238815	14	36,741,794	–	− 0.00223	1.4E-09	–	–	− 0.21188	1.68E-05

In addition to the two overlapping CpG sites, 124 common differentially methylated CpG sites were identified from the two EWASs of the different COVID-19 status test methods (Supplementary file 2, Table S5). The effect sizes between the two EWASs (PCR-based and antibody-based methods) were strongly consistent (*R* = 0.88). Among the results, cg05655377 on chromosome 4 was one of the hypermethylated top effect sites in both EWASs. Cg11586124 and cg19722371 on chromosome 12 were the hypomethylated top effect sites common to both EWASs. Cg19722371 was annotated to gene *DARM1*.

Comparing the findings of the different exploration approaches with the EWAS catalog, none of the CpG sites had been previously reported in relation to COVID-19. Enrichment analysis showed that both GO and KEGG terms returned no statistically significant results.

### Sensitivity analysis

Given that smoking status has a great impact on DNAm patterns, regression models with the same covariates plus smoking status were used in the analysis. Using three models, with phenotypes indicating anti-SARS-CoV-2 antibody levels, COVID-19 status based on antibody testing, and COVID-19 status based on PCR testing, we identified 15, 130, and 394 differentially methylated CpG sites after bacon correction at FDR < 0.05, respectively (Supplementary file 2, Table S6-S8). Among these CpGs, 15 (100%), 129 (99.2%), and 389 (98.7%) out of them appeared in the main analysis and all of them had a consistent direction, showing that the primary findings was robust to smoking status.

By removing participants without clear blood sample collection dates and with short intervals between PCR test and blood collection, we eliminated 11 persons and analyzed the remaining subset using regression models with the same covariates. The EWAS results can be found in Supplementary file 2, Table S9-S11. Overall, 37 (88%) differentially methylated CpGs in the first model (association with anti-SARS-CoV-2 antibody levels), 158 (91.8%) CpGs in the second model (COVID-19 status based on antibody testing), and 443 (88.2%) CpGs in the third model (COVID-19 status based on PCR testing), were also discovered in the main analysis and the effect directions were consistent. Additionally, 3 common differentially methylated CpGs (cg04900672, cg17126990, cg25483596) were identified, and 2 of them (cg17126990, cg25483596) were the main findings in the primary analysis, indicating that the association of the two CpGs with antibody levels and case status described in main results were robust to variations in the estimated intervals from infection to blood collection.

## Discussion

We investigated DNAm changes with anti-SARS-CoV-2 antibody levels and compared DNAm patterns between mild and asymptomatic patients and healthy individuals after 7 months of infection in a household setting. Overall, numerous differentially methylated CpG sites were identified, demonstrating that DNAm changes of post-infection are associated with antibody levels and that patients with mild and asymptomatic symptoms have DNAm patterns that differ from never infected controls. Two common CpG sites, namely cg17126990 (*AFAP1L2*) and cg25483596 (*PC*), showed positive associations with both antibody levels and case status.

*AFAP1L2* enables SH3 domain binding activity and protein tyrosine kinase activator activity and is involved in the positive regulation of the epidermal growth factor receptor signaling pathway, associated with vesicoureteral reflux and cartilage cancer [36]. Studies showed that *AFAP1L2* was upregulated in A549-ACE2 cells [37], human nasal epithelial cells [38], and mouse kidneys after SARS-CoV-2 infection [39]. According to data from blood cell lines, cg17126990 overlapped both a DNasel hypersensitivity and an open chromatin regions, which suggests this CpG is involved in the transcription process [40]. Additionally, a genome browser search showed that both transcription factors ELAVL1 and PABPC1 bind the region containing cg17126990 [40]. ELAVL1 is highly expressed in cancer cells and is also involved in inflammation by regulating mRNA stability, splicing, and translation [41]. Lu et al. [42] found that *ELAVL1* was upregulated in COVID-19 patients and can effectively predict SARS-CoV-2 infection with other six m6A-related genes. The second identified CpG site, cg25483596, is annotated to gene *PC*. *PC* is a protein-coding gene involved in gluconeogenesis, lipogenesis, insulin secretion, and synthesis of the neurotransmitter glutamate [36]. SARS-CoV2 infection was found to alter host cell metabolism by upregulating PC activity to increase carbon entry into the TCA cycle [43]. Analyses in cell lines show that cg25483596 overlapped regions with histone modifications, weak enhancers, and weakly transcribed regions in blood cells [40].

We also found different DNAm patterns between persistent health restriction cases and recovered cases. Cg27139398 (annotated to *ADAM22*) and cg23815787 (annotated to *UBE2N*) were two of the top 5 negatively correlated CpG sites. EI-Agnaf et al. [44] discovered that *ADAM22* may contribute to neurological complications in post-severe COVID-19 patients, while *UBE2N* was downregulated in SARS-CoV-2 late-stage infection in human blood samples [45].

By using publicly available data, cg02942825 (annotated to *GIPR*), cg10118093 (annotated to *PIK3C2B*), and cg26589785 (annotated to *ITPKA*) were found to be reported cis-meQTLs and cis-eQTMs, indicating that the CpG sites are influenced by genetic variants nearby, and might potentially mediate the association between genetic variants and downstream phenotype. *GIPR*, *PIK3C2B*, and *ITPKA* are all protein-coding genes related to multiple traits [46]. Previous studies showed an association between *GIPR* and C-reactive protein levels [47] and *ITPKA* was found to be related to respiratory system diseases [48], while *PIK3C2B* was reported to be associated with lung function [49, 50].

Lee et al. [16] investigated the DNAm profile between COVID-19 cases and controls after 3 months post-infection; they report DNAm patterns in long COVID patients, highlighting the immune response associated gene *IFI44L* as their main finding. We found no overlapping CpG sites when comparing our results with those of Lee et al. One reason might be that we have only mild and asymptomatic cases, while Lee et al. had a study population with severe symptoms. Another reason could be the follow-up time post-infection for the DNAm measurement, as the epigenetic profile likely further changes after an additional four month post-infection. There are also no common CpG sites between our findings and COVID-19-related studies reported in the EWAS catalog, indicating that mild cases could have a different DNAm pattern at 7 months of post-infection.

There are some strengths of our study. Different COVID-19 case definitions were used in this study, providing a more comprehensive estimation. To our knowledge, this is the first study to focus on asymptomatic and mild COVID-19 cases with a relatively long follow-up after infection 7 months in a household setting. In addition, the GEE model takes the correlation of DNAm patterns within household members into account, leading to a reliable and robust estimate.

There are also a few limitations. The study population was 14 years and older, living in private households in the Munich area, which may limit its generalizability. We also found differentially methylated CpG sites between cases with self-reported health restriction cases and those fully recovered, yet these results are limited by the instrument used to determine health restrictions and potential misclassification of the groups. We used publicly available data to identify meQTLs and eQTMs, which might not necessarily reflect associations in our study population.

## Conclusions

We investigated DNAm changes in asymptomatic, mild cases, and healthy individuals 7 months post-SARS-CoV-2 infection. Cg17126990 (annotated to *AFAP1L2*) and cg25483596 (annotated to *PC*) were the common CpG sites identified to be differentially methylated across the different study approaches, thus suggesting their relevance in the aftermath of COVID-19. Both CpGs have been reported to be involved in molecular pathways associated with SARS-CoV-2 infection. These findings may be useful for further understanding the molecular mechanism after SARS-CoV-2 infection in mild and asymptomatic individuals.

## Data availability 

Data at individual level are not available due to protection of data privacy of our study subjects. However, data are accessible subject to data protection regulations upon reasonable request to the KoCo19 investigators. Requests will be scientifically reviewed following the cohort’s review process.

## Supplementary Information

Additional file1 (DOCX 206 KB)

Additional file2 (XLSX 174 KB)

## Abbreviations

COVID-19: Coronavirus disease 2019
SARS-CoV-2: Severe acute respiratory syndrome coronavirus 2
DNAm: DNA methylation
ACE2: Angiotensin-converting enzyme 2
TMPRSS2: Type 2 transmembrane serine protease
CpG: Cytosine-phosphate-guanine
EWAS: Epigenome-wide association study
KoCo19: Prospective COVID-19 cohort Munich
PCR: Polymerase chain reaction
QN: Quantile normalization
EDTA: Ethylenediaminetetraacetic acid
GEE: Generalized estimating equation
IQR: Interquartile range
GO: Gene ontology
KEGG: Kyoto Encyclopedia of Genes and Genomes
FDR: False discovery rate
meQTL: Methylation quantitative trait loci
eQTM: Expression quantitative trait methylation
PC: Pyruvate carboxylase
BMI: Body mass index
