# Supplementary material for: Differential DNA methylation 7 months after SARS-CoV-2 infection
Source: Clin Epigenetics. 2025 Apr 18;17:60. doi: 10.1186/s13148-025-01866-4 (PMC12008906; doi:10.1186/s13148-025-01866-4)
Supplement: Supplementary file 1 — Additional file1 (DOCX 206 KB) [file 13148_2025_1866_MOESM1_ESM.docx]

Additional file 1

Supplementary Figures:


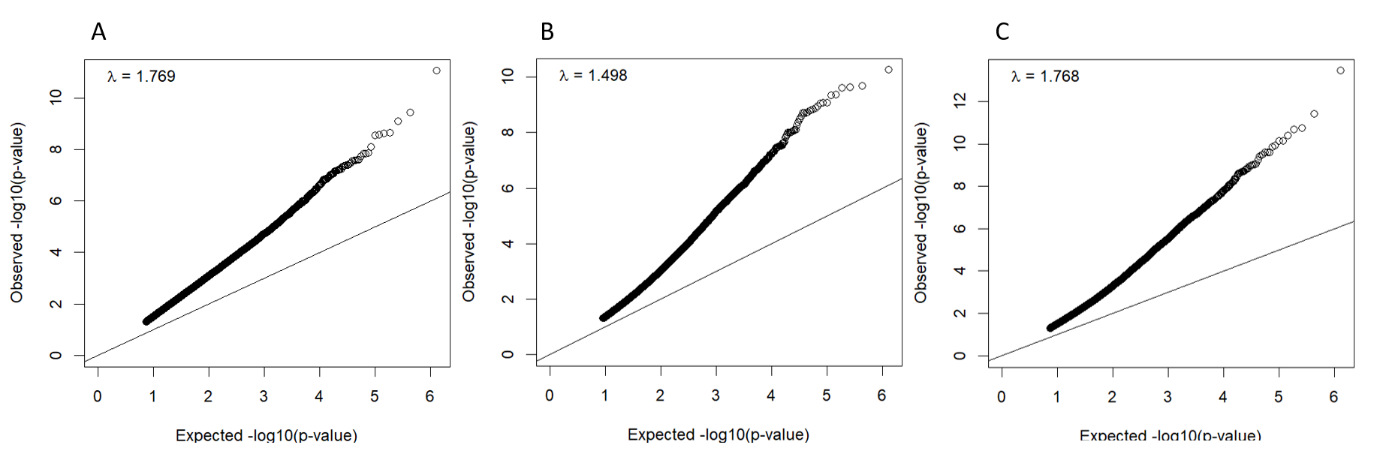


Supplementary Figures 1: **A** QQ plot of the EWAS results for anti-SARS-CoV2 antibody level (lambda: 1.769). **B** QQ plot of the EWAS results between COVID-19 cases and controls based on antibody testing (lambda: 1.498). **C** QQ plot of the EWAS results between COVID-19 cases and controls based on PCR testing (lambda: 1.768).


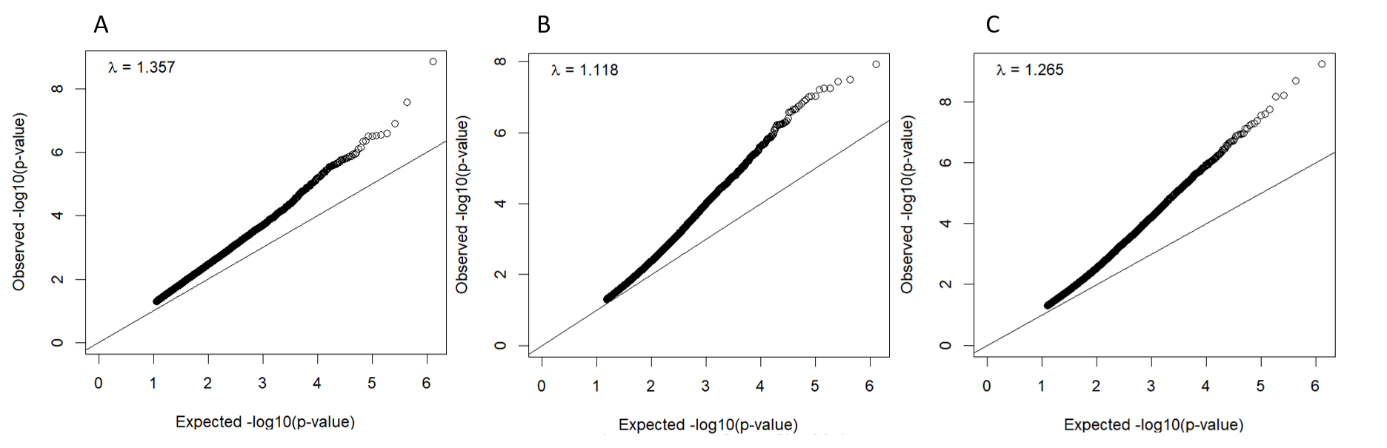


Supplementary Figures 2: **A** QQ plot of the EWAS results for anti-SARS-CoV2 antibody level after bacon correction (lambda: 1.357). **B** QQ plot of the EWAS results between COVID-19 cases and controls based on antibody testing after bacon correction (lambda: 1.118). **C** QQ plot of the EWAS results between COVID-19 cases and controls based on PCR testing after bacon correction (lambda: 1.265).
